# Cornelia de Lange Syndrome Accompanied by Cholelithiasis and Nephrolithiasis: A Case Report

**DOI:** 10.3390/children11121433

**Published:** 2024-11-26

**Authors:** So Yoon Choi, Yoo-Rha Hong, Chi-Eun Oh, Jung Hyun Lee

**Affiliations:** 1 Departments of Pediatrics, Kosin University Gospel Hospital, Kosin University College of Medicine, Busan 49267, Republic of Korea; 2 Departments of Pathology, Kosin University Gospel Hospital, Busan 49267, Republic of Korea

**Keywords:** Cornelia de Lange syndrome, *NIPBL*, cholelithiasis, nephrolithiasis

## Abstract

Cornelia de Lange syndrome (CdLS) is a rare genetic disorder characterized by a distinctive facial appearance, growth/cognitive retardation, developmental delay, skeletal malformation, hypertrichosis, and other abnormalities. Patients with mild CdLS have less severe phenotypes, while retaining representative facial features. Mutations in the genes *NIPBL*, *SMC1A*, *SMC3*, *HDAC8*, and *RAD21* have been associated with CdLS, with mutations in *NIPBL* accounting for approximately 60% of cases. Herein, we present a case of CdLS accompanied by cholelithiasis and nephrolithiasis. A 9-year-old Korean boy presented with vomiting and abdominal pain. Abdominal ultrasonography revealed several gallstones and renal stones. Extracorporeal shock wave lithotripsy failed; therefore, cholecystectomy and nephrolithotomy were performed. Postoperative stone composition analysis revealed calcium oxalate as the primary component. CdLS was suspected based on the characteristic appearance and physical examination, with genetic testing confirming an *NIPBL* gene mutation. Simultaneous CdLS, cholelithiasis, and nephrolithiasis requires careful management and treatment tailored to each patient’s specific needs and challenges.

## 1. Introduction

Cornelia de Lange syndrome (CdLS) is a rare genetic disorder associated with a wide spectrum of developmental and physical abnormalities. It is characterized by a distinctive facial appearance, growth retardation, cognitive impairment, developmental delay, skeletal malformations, hypertrichosis, and a variety of other systemic anomalies, including gastrointestinal and genitourinary abnormalities [1,2,3]. The clinical presentation of CdLS exhibits significant phenotypic variability, ranging from mild to severe [4]. In severe cases, the diagnosis can often be made in the early neonatal period due to pronounced physical and developmental anomalies. However, in milder phenotypes, diagnosis may be delayed because of subtler clinical features [5,6]. Patients with milder forms of CdLS often have less severe growth, cognitive, and limb involvement but still retain characteristic facial features that are consistent with the syndrome [5,7]

CdLS is primarily an autosomal dominant disorder caused by mutations in genes that regulate chromosomal cohesion and transcriptional control. Mutations in Nippe-B-like (*NIPBL*), Structural maintenance of chromosomes 1A (*SMC1A*), Structural maintenance of chromosomes 3 (*SMC3*), and other genes involved in the cohesin pathway, such as *RAD21*, *HDAC8*, and *BRD4*, have been implicated in CdLS [8,9,10,11]. Mutations in the *NIPBL* gene account for approximately 60% of cases, making it the most commonly affected gene in CdLS [9]. While many cases result from de novo mutations, inherited cases have been documented, albeit rarely [12]. The genetic and phenotypic variability of CdLS underscores the importance of precise genetic and clinical evaluation in establishing a diagnosis.

CdLS was first described in 1933 by Cornelia de Lange, a Dutch pediatrician [13]. In Korea, the first documented case was reported by Moon et al. in 1967 [14]. Since then, several additional cases have been reported, primarily focusing on classical features such as facial dysmorphism, limb anomalies, and growth delays. During the 1990s, two cases with limb malformations, including syndactyly, were described in Korean patients. However, to date, no cases of CdLS associated with cholelithiasis or nephrolithiasis have been reported in the Korean population.

Herein, we report a unique case of CdLS in a 9-year-old Korean male patient presenting with cholelithiasis and nephrolithiasis. This case highlights the expanding phenotypic spectrum of CdLS and underscores the need for a comprehensive approach to diagnosing and managing CdLS, particularly in cases with atypical manifestations.

## 2. Case Presentation

A 9-year-old Korean boy presented to a local clinic with non-bilious vomiting and abdominal pain lasting 3 days. Abdominal ultrasound revealed several gallstones and renal stones, and he was subsequently referred to our hospital for further evaluation and treatment.

**Figure 1:**
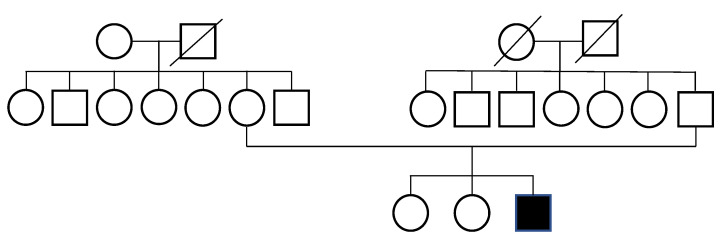
Family pedigree chart.

The patient was born at term, with a birth weight of 3.2 kg (38th percentile). At presentation, he had a height and weight of 110 cm (<3rd percentile) and 15 kg (<3rd percentile), respectively. His head circumference was 48 cm (<3rd percentile). The family history (Figure 1) and medical history were unremarkable, with the patient’s siblings not exhibiting features of CdLS. However, the patient attended a special school due to developmental and intellectual disabilities.

**Figure 2:**
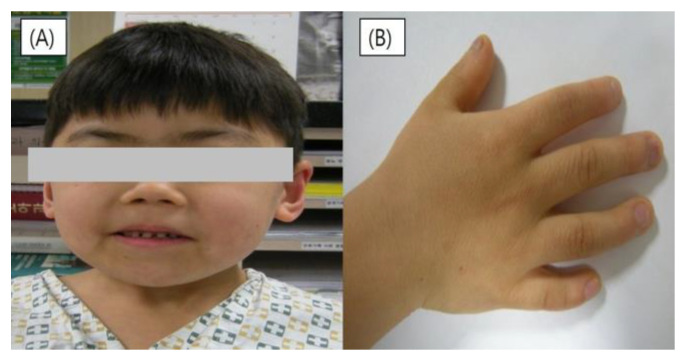
Clinical characteristics of patients with Cornelia de Lange syndrome: (**A**) Facial morphology of patients (synophrys, dark eyebrows, a short glabella, mildly lifted nostrils, downturned mouth angles, and a thin lower lip). (**B**) Clinodactyly of right hand.

The patient had synophrys (bushy eyebrows meeting in the midline), with dark eyebrows and long curly eye lashes, a short glabella, low back hairlines, mildly lifted nostrils, downturned mouth angles, and a thin lower lip (Figure 2). His hands and feet were small, and his distal interphalangeal joints were bent, indicating clinodactyly. Other findings supporting the diagnosis of CdLS included microcephaly, micropenis, and general hypertrichosis.

Laboratory tests revealed the following: hemoglobin, 11.8 g/dL; white blood cells, 6770/mm^3^; platelets, 236,000/mm^3^; blood urea nitrogen/creatinine 17/0.4 mg/dL; sodium, 141 mEq/L; potassium, 3.9 mEq/L; calcium, 9.7 mg/L; phosphorus, 4.6 mg/L; and aspartate aminotransferase/alanine aminotransferase (AST/ALT) 28/17 IU/L. These were all within the normal range.

**Figure 3:**
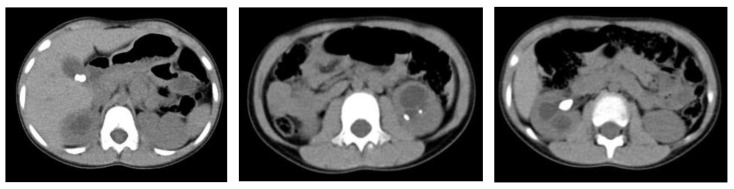
Abdominal computed tomography shows a gallstone and renal stones.

An abdominal X-ray showed several radio-opaque calcifications of varying sizes in the right upper mid-abdomen. Abdominal computed tomography (CT) revealed a gallstone measuring 1.2 × 0.8 cm, three renal calculi measuring 1 × 1 cm, 0.6 × 0.3 cm, and 2 × 2 cm in the right kidney, and hydronephrosis (Figure 3). The spot urine test revealed no specific findings, and the urine culture test was negative. The 24 h collected urine test revealed the following: calcium/creatine ratio, 0.09 (normal range < 0.2); oxalate/creatine ratio, 0.015 (normal range < 0.1); uric acid/creatine ratio, 1.14 (normal range 0.594–2.378); and citrate/creatine ratio, 0.18 (normal range < 0.51), with no abnormalities.

**Figure 4:**
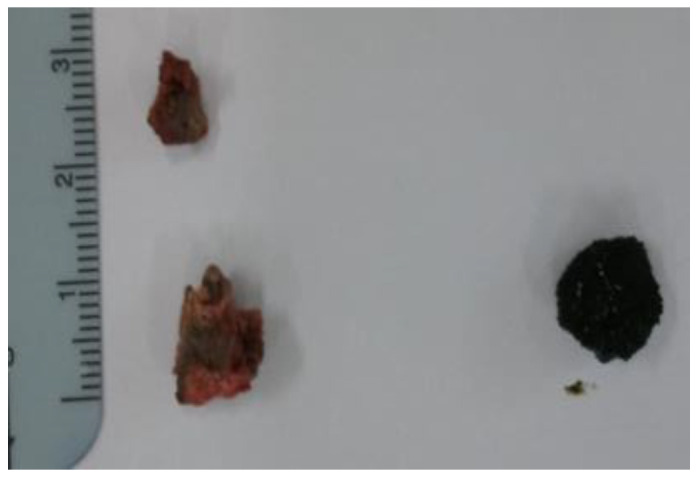
Renal pigment stones.

The patient’s symptoms persisted, and extracorporeal shock wave lithotripsy resulted in no improvements. Therefore, cholecystectomy and nephrolithotomy were performed in collaboration with the Department of Hepatobiliary surgery and Urology. Post-operative stone composition analysis revealed that the gallstone and kidney stones were composed of calcium oxalate, which is the most common type of pigment stone observed in pediatric patients (Figure 4).

## 3. Genetic Analysis

**Figure 5:**
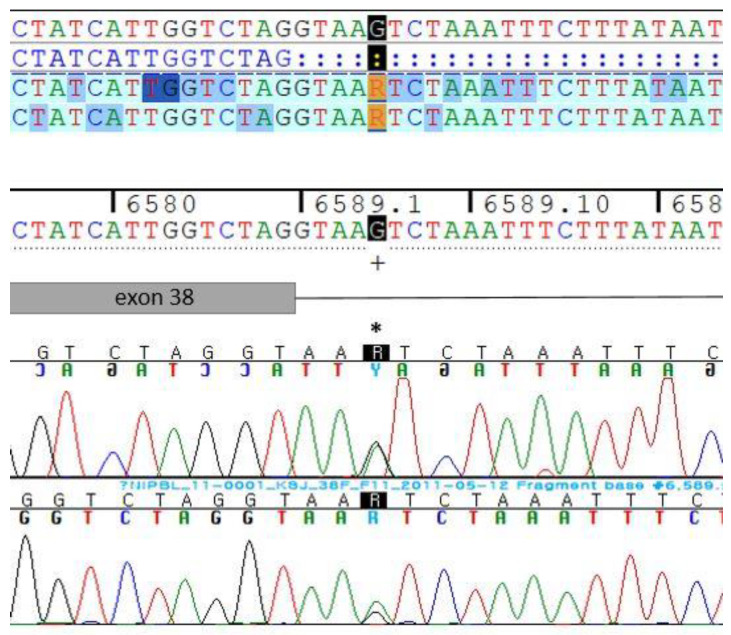
*NIPBL* gene sequencing.

CdLS was suspected based on the patient’s characteristic appearance and the results of the physical examination, leading to subsequent genetic testing. Due to the unavailability of a comprehensive CdLS gene panel or whole-exome sequencing (WES) in Korea at the time, testing was limited to the *NIPBL* gene, the most commonly implicated causal gene in CdLS. Genetic analysis revealed a previously identified G-to-A mutation in the c.6589+5th nucleotide sequence of intron 38 (Figure 5). All exons (protein-coding regions) and adjacent exon–intron boundaries (up to 10 nucleotides) of the *NIPBL* gene were sequenced. However, other CdLS causal genes (*SMC1A*, *SMC3*, *HDAC8*, and *RAD21*) and noncoding regions (e.g., untranslated regions (UTRs), deeper intronic regions) were not analyzed, leaving some uncertainty about other potential causal variants. Additionally, copy number variants (CNVs), which are not typically analyzable with conventional sequencing methods, were not assessed. Parental testing, which is typically recommended to confirm whether the mutation is de novo, was not performed because the parents declined genetic testing. This omission limits our ability to conclusively determine the origin of the mutation.

## 4. Discussion

This case presents a unique instance of CdLS in a pediatric patient who was diagnosed with both cholelithiasis and nephrolithiasis. Although no direct pathophysiological association between CdLS and these conditions has been established, the rarity of their simultaneous occurrence in a CdLS patient raises questions about potential underlying genetic or syndromic factors.

CdLS is primarily inherited in an autosomal dominant or X-linked dominant manner. The possibility of an autosomal recessive inheritance pattern remains uncertain, with some suggesting it may result from mosaicism [9]. Among CdLS-related genes, mutations in *NIPBL* are the most common, accounting for 60–70% of cases. *NIPBL* encodes delangin, a protein critical for sister chromatid cohesion, regulation of long-range enhancer–promoter interactions, and DNA repair during the G2 phase of the cell cycle [15].

In this case, chromosomal analysis showed a 46,XY karyotype, and genetic testing of the *NIPBL* gene revealed a c.6589+5 G>A splicing mutation in intron 38. This mutation has been reported in prior studies and cited as pathogenic in the literature [16,17]. However, under ACMG/AMP guidelines, this mutation is currently classified as a variant of uncertain significance (VUS). The mutation has not been observed in large population databases, suggesting it is rare, but there are insufficient functional data to establish its pathogenicity conclusively. Additionally, splicing prediction tools suggest that the mutation is unlikely to significantly impact normal gene splicing. However, if future studies confirm that this mutation is de novo, its classification could be upgraded to likely pathogenic under ACMG criteria.

This case highlights the importance of combining clinical findings with detailed genetic analysis to achieve an accurate diagnosis and to classify genetic variants appropriately. In regions where comprehensive gene panels or advanced sequencing methods are unavailable, targeted single-gene testing of commonly implicated genes, such as *NIPBL*, remains valuable. Further functional studies and familial testing, including confirmation of de novo status, are crucial for reclassifying VUS findings and improving their clinical interpretation.

This patient presented with a milder CdLS phenotype, which can delay diagnosis due to subtle physical features. This emphasizes the importance of thorough clinical examinations, especially of facial and physical characteristics, even in the absence of overt symptoms. Genetic testing played a critical role in confirming the diagnosis. Although mutations in NIPBL are established causes of CdLS, their potential role in predisposing patients to biliary or renal stones remains speculative and warrants further investigation.

Gallstones, defined as stones in the gallbladder, are exceedingly rare in pediatric patients and are generally associated with predisposing factors such as hemolytic anemia, metabolic disorders, infections, anatomical abnormalities, and bile stasis [18]. Recently, ultrasound examinations have become more common in pediatric patients, leading to an increased detection of gallstones. Pediatric gallstones typically differ from those in adults, with pigment gallstones being more common than pure cholesterol or bile stones. The etiology of gallstone formation is multifactorial and involves various contributing factors [18,19,20,21].

However, the relationship between cholelithiasis and CdLS remains poorly understood, with no well-established risk factors identified for gallstone formation in these patients. This case presents an unusual occurrence of gallstones in a patient with CdLS, raising the possibility of a unique underlying pathophysiological mechanism. The gallstones in this patient were identified as calcium bilirubinate stones, primarily composed of polymerized or monomeric forms of calcium bilirubinate. These stones likely formed due to increased precipitation and aggregation of insoluble unconjugated bilirubin in the bile, or from chronic infection in the bile duct system, where bacterial beta-glucuronidase deconjugates bilirubin within the bile [22].

Potential factors contributing to gallstone formation in CdLS could include the syndrome’s association with gastrointestinal motility issues, leading to bile stasis, or metabolic alterations that increase the risk of bilirubin precipitation. Recurrent infections or a predisposition to altered hepatic or biliary function may also play a role. While these hypotheses require further investigation, understanding these mechanisms could provide significant insights into the early detection and management of gallstones in CdLS patients. Considering the lack of robust data, targeted imaging techniques such as routine abdominal ultrasound could be considered for CdLS patients, particularly those presenting with gastrointestinal symptoms or risk factors for gallstone formation. However, the decision to implement routine screening should balance the benefits and risks, as imaging may not be necessary for all patients. This case underscores the importance of continued research into the prevalence and pathophysiological mechanisms of cholelithiasis in genetic syndromes like CdLS. Further studies may help clarify these associations and inform clinical guidelines, ultimately improving care for this unique patient population.

Treatment options for gallstones include cholecystectomy (surgical removal of the gallbladder) and conservative management [23]. The choice between these approaches depends on factors such as the child’s age and the severity of symptoms. Cholecystectomy is the preferred treatment for symptomatic cases, while surgical removal in young or asymptomatic children is a subject of debate, with a preference for conservative management. The prognosis following surgical removal of gallstones is generally favorable, and indeed, no complications were observed after the surgical procedure in this patient.

Pediatric urinary stones often occur in association with structural abnormalities of the genitourinary system, metabolic abnormalities, and urinary tract infections [24]. Since children with urinary stones have a high frequency of metabolic abnormalities and a high risk of recurrence, it is necessary to check for metabolic abnormalities through blood and urine tests [25]. Stones obtained from patients are generally analyzed for composition to identify basic data for treatment policies to prevent recurrence. In previous studies, the primary component of stones in 75–80% of cases was calcium oxalate, 10–20% were infected stones (struvite), pure uric acid stones accounted for 5%, while calcium phosphate stones were more common than in adults at 9% [26,27]. Our patient was also confirmed to have the most common calcium oxalate stone.

Genitourinary abnormalities are common in patients with CdLS, with the most common being structural abnormalities of the kidneys and urinary tract, such as the absence or poor cortico-medullary differentiation, pelvic dilatation, vesicoureteral reflux, small kidneys, solitary renal cysts, and renal ectopia [28]. This can lead to decreased kidney function and recurrent infections. In our patient, we identified renal calculi along with hydronephrosis. Cases of CdLS accompanied by renal calculi have been rarely reported [29]. Although reports of CdLS patients with actual kidney stones are rare, kidney stones may occur due to anatomical abnormalities in the kidneys; as such, it is necessary to closely check for structural problems in the kidneys and to determine whether kidney stones are present in CdLS patients.

One previous study reported the autopsy findings of two CdLS patients, showing that both patients exhibited a pyramidal shape of the liver and kidneys, which was suggested to potentially manifest as pathological symptoms prone to biliary and renal pelvis obstruction and kidney stone formation in the later stages [30].

While this case provides new insights into potential complications in CdLS, further studies are needed to explore possible genetic or anatomical predispositions to biliary and renal stones in CdLS. Careful management of concurrent conditions like cholelithiasis and nephrolithiasis in CdLS patients is crucial, given the complexities these diseases present. This report adds to the body of knowledge by documenting a rare case of simultaneous renal and gallbladder stones in a CdLS patient, emphasizing the importance of individualized care for such patients.

## 5. Conclusions

This report presents a rare case of concurrent cholelithiasis and nephrolithiasis in a patient with CdLS, a combination that is uncommon in CdLS patients who exhibit diverse clinical symptoms. This case provides new insights into the complex clinical manifestations associated with CdLS, highlighting the importance of careful monitoring and treatment tailored to the unique needs of these patients. Additionally, it underscores the need for further research to investigate any genetic or pathological links between CdLS and the development of renal calculi and gallstones.

## Data Availability

The data presented in this study are available on request from the corresponding author due to privacy.
